# Supplementary material for: Health technology assessment system in Switzerland: Current state, gaps, and prospects for improvement
Source: PLOS Glob Public Health. 2026 Mar 6;6(3):e0005848. doi: 10.1371/journal.pgph.0005848 (PMC12965548; doi:10.1371/journal.pgph.0005848)
Supplement: Attachment — Submitted filename: Plos HTA Switzerland Paper_Response-Table-2025-.docx [file pgph.0005848.s004.docx]

**Reviewer Comments for Authors from PLOS Global Public Health Decision**

Health Technology Assessment System in Switzerland: Current State, Gaps, and Prospects for Improvement

Mohammed Alkhaldi*1,2,3, Rima Kachach4, Line Enjalbert1, Aisha Al Basuoni6, Sara Ahmed1

PGPH-D-25-02848

Resubmission to PLOS Global Public Health Decision

We’d like to thank the reviewers for their comments, which have substantially improved the quality of our manuscript. Below, we present how we’ve addressed each comment.

Journal Requirements:

1. Please amend your detailed online Financial Disclosure statement. This is published with the article. It must therefore be completed in full sentences and contain the exact wording you wish to be published.

a) State the initials, alongside each funding source, of each author to receive each grant. For example: “This work was supported by the National Institutes of Health (####### to AM; ###### to CJ) and the National Science Foundation (###### to AM).”

b) State what role the funders took in the study. If the funders had no role in your study, please state: “The funders had no role in study design, data collection and analysis, decision to publish, or preparation of the manuscript.”

For more information, please go to our submission guidelines:
<https://journals.plos.org/globalpublichealth/s/submission-guidelines#loc-financial-disclosure-statement>

Authors' response: A revised funding statement has been provided, including the required information. This statement now reads as follows:

“This study was supported by McGill University, Montreal, Canada, in the form of a grant (MITACS – 259863, CHIRS – 259844) and by the Lindsay Foundation Grant at CIUSSS Centre-Sud-de-l’Ile-de-Montréal in the form of a grant awarded (CIUSS 2021). Both grants were awarded to M. Alkhaldi, the corresponding author of this study, whose specific roles are articulated in the “Author Contributions” section. The funders had no role in study design, data collection and analysis, decision to publish, or preparation of the manuscript."

2. Please ensure that the funders and grant numbers match between the Financial Disclosure field and the Funding Information tab in your submission form. Note that the funders must be provided in the same order in both places as well.

Author's response: This has been checked and reflected.

3. Please update your online Competing Interests statement. If you have no competing interests to declare, please state: “The authors have declared that no competing interests exist.”

Author's response: This statement has been inserted in the manuscript and submission system.

4. In the online submission form, you indicated that “Anonymized data used in the current study are available upon request from the authors. Anonymized data used in the current study are available upon request from the authors.”.

All PLOS journals now require all data underlying the findings described in their manuscript to be freely available to other researchers, either 
a) In a public repository, 
b) Within the manuscript itself, or 
c) Uploaded as supplementary information.

This policy applies to all data except where public deposition would breach compliance with the protocol approved by your research ethics board. If your data cannot be made publicly available for ethical or legal reasons (e.g., public availability would compromise patient privacy), please explain your reasons by return email and your exemption request will be escalated to the editor for approval. Your exemption request will be handled independently and will not hold up the peer review process, but will need to be resolved should your manuscript be accepted for publication. One of the Editorial team will then be in touch if there are any issues.

For further assistance, you may go to: <http://journals.plos.org/globalpublichealth/s/data-availability>

Authors' response: An updated statement of data availability, including the necessary information requested by the journal, has been placed in the manuscript and the submission system. It now reads as follows:

**“Data availability statement:** The data contains identifying and potentially sensitive information related to participating organizations and experts. Data sharing is subject to restrictions imposed by the McGill Research Board Ethics Office in Canada. Access to the data may be requested from the corresponding author or the hosting institution upon reasonable request. Requests for data access can be directed to: The Faculty of Medicine and Health Sciences Research Ethics Board (McGill IRB), McGill University: Georgia Kalavritinos, Ethics Review Administrator, REB 1, 2, 3, georgia.kalavritinos@mcgill.ca.”

5. Please provide separate main figure files in .tif or .eps format only and ensure that all files are under our size limit of 10MB.

For more information about how to convert your figure files please see our guidelines: <https://journals.plos.org/globalpublichealth/s/figures>

Authors’ response: all figures have been changed and uploaded in tif format.

6. We have noticed that you have a list of Supporting Information legends in your manuscript. However, there are no corresponding files uploaded to the submission. Please upload them as separate files with the item type 'Supporting Information'.

Authors’ response: The supporting information legends have been revised.

7. If the reviewer comments include a recommendation to cite specific previously published works, please review and evaluate these publications to determine whether they are relevant and should be cited. There is no requirement to cite these works unless the editor has indicated otherwise.

Authors’ response: all required citations or references have been inserted.

Review Comments to the Author

Please use the space provided to explain your answers to the questions above. You may also include additional comments for the author, including concerns about dual publication, research ethics, or publication ethics. (Please upload your review as an attachment if it exceeds 20,000 characters)

| **Reviewers’ comment** | | **Description of revisions** | **Location of revisions** |
| --- | --- | --- | --- |
| **Section A: Reviewer 1 Comments** | | | |
| General: This study is very timely as with the HTAR starting its implementation in 2025, though EEA countries are not part of the Coordinating Group, they will need to continue to be part of the evolving HTA landscape in Europe and I think therefore a review of the HTA system is very helpful for the country. That said it maybe helpful to mention the HTAR in the manuscript in the abstract and definitely in the introduction otherwise this is looking very dated as the landscape some 2-3 years back. Seeing the dates of the research – this is clear that it is some years old, but then the context and introduction & discussion should reflect the HTA scenario in 2025.  https://health.ec.europa.eu/health-technology-assessment/implementation-regulation-health-technology-assessment_en  I think the study is very good but needs better and improved reporting and putting into context for it to be really valuable and helpful. | | Thank you for this very useful suggestion. The authors revised the manuscript and added two relevant paragraphs to the abstract and intro sections.  Below paragraph has been added to abstract:  *“HTA Regulations (HTAR 2021/2282) is the EU’s new framework for joint clinical assessments. While Switzerland is not part of it, it maintains a distinct HTA system that often aligns with EU requirements and reflects its unique healthcare governance model, with structured processes led by the Federal Office of Public Health (FOPH), despite lacking a comprehensive national framework like those in the UK or Canada.”*  This paragraph has been added to the intro section:  *“HTAR (HTA Regulation, 2021/2282) is the new EU-wide framework that governs how Member States will jointly conduct clinical assessments of health technologies. While Switzerland is not part of the HTAR, its national regulations often align closely with EU requirements.  Switzerland has its own established HTA program, and this national program is distinct from the new EU system. The Swiss HTA landscape reflects these differences in several ways, shaped by the country’s unique healthcare governance model.”* | **Abstract and intro** |
| **Introduction:** | |  |  |
| • EunetHTA initially started in 2006 as a framework 7 EU funded project 2006-2008 then there was a transition period for a year and then as you say Joint Action 1 was from 2010. So currently this isn’t clear – or reads incorrect, as I worked for EunetHTA on behalf of UK from 2008, I think this needs to be rectified. https://www.healthinformationportal.eu/european-initiative/european-network-health-technology-assessment | | Thank you for your comment and for sharing your firsthand experience. We appreciate the clarification. The authors have revised this section and reformulated the paragraph, which now reads as follows, based on your suggestion:  *“EUnetHTA initially started in 2006 as a Framework 7 European Union-funded project (2006–2008), followed by a one-year transition period, after which Joint Action 1 began in 2010. This Joint Action established EUnetHTA as a sustainable and effective HTA collaboration across Europe, designed to provide added value at the local, national, and EU levels.”* | **intro** |
| • EuneHTA21 also officially shut down in September 2023 in anticipation of the HTAR – so the tense needs to be changed e.g. “The EUnetHTA is composed of 35  government-appointed organizations from 24…” it should say “EUnetHTA was composed of …” | | Thanks for this comment. The authors have amended this sentence in the revised manuscript, and it now reads as follows:  *“EUnetHTA was composed of …”* | **intro** |
| • The sentence here is again not a 1005 clear “Although there is some standardization in national HTA systems across Europe, notable differences remain in both process and methodology” With the HTAR now there is joint clinical assessment – other domains economic, other considerations etc and actual appraisal or decision making at done differently in different countries but it is important to clarify that 27 EU countries now have to follow the same process for clinical assessments and have joint clinical assessments for oncology, ATMPs already with selected medical devices soon starting then orphan drugs in 2028 and everything from 2030 – this is very important and is missing from the context. | | Thanks for this useful suggestion. The authors have revised this paragraph and reformulated it, providing further explanation supported by a new reference, as requested by the reviewer. The paragraph now reads as follows:  *Although there is some standardization in national HTA systems across Europe, notable differences remain in both process and methodology. HTAR (HTA Regulation, 2021/2282) is the new EU-wide framework that governs how Member States will jointly conduct clinical assessments of health technologies. While Switzerland is not part of the HTAR, its national regulations often align closely with EU requirements.  Switzerland has its own established HTA program, and this national program is distinct from the new EU system. The Swiss HTA landscape reflects these differences in several ways, shaped by the country’s unique healthcare governance model. With the HTAR, there is now a joint clinical assessment, while other domains, such as economic evaluations and decision-making processes, are handled differently across countries. It is important to note that all EU countries now follow the same process for clinical assessments, with joint assessments already in place for oncology and Advanced Therapy Medicinal Products, expanding soon to selected medical devices, orphan drugs in 2028, and all other therapies by 2030 (new ref).”* | **Intro** |
| • There is also some strange phrasing – e.g. “However, the statutory health insurance system, including the benefit package, is managed centrally at the federal level. Switzerland has the second-highest life expectancy (82.8 years) in Europe, and healthy life expectancy exceeds the EU average by several years” – how and where the benefit package is managed shouldn’t be in contrast with the life expectancy – so the use of however seems strange. | | The authors have revised and amended this paragraph in the revised manuscript according to the reviewer’s comment, with the removal of “however”. The paragraph now reads as follows:  *“The statutory health insurance system and its benefit package in Switzerland are managed centrally at the federal level, with insurers providing coverage according to federal rules. This system contributes to Switzerland’s high life expectancy (82.8 years), with healthy life expectancy exceeding the EU average (7).”* | **Intro** |
| • Clearly the insurance and reimbursement system is fragmented and complex in Switzerland so I appreciate the authors trying to describe it – however it isn’t completely clear what is done by national level and what by cantons and how they interplay – maybe a figure could be helpful here. | | Thank you for this helpful suggestion. The authors have revised the introduction and incorporated this important point.  The authors have added the following paragraph, which clarifies the responsibilities at both levels and explains how they interrelate:  *“In Switzerland, the insurance and reimbursement system is notably fragmented and complex. The federal government centrally regulates statutory health insurance, including defining the mandatory benefit package, setting national reimbursement rules, and establishing uniform requirements for insurers. The cantons are responsible for implementing these federal regulations, financing and managing hospitals, and overseeing regional service delivery. The two federal and cantonal levels operate through a shared governance structure: federal legislation defines the scope of coverage, while the cantons ensure its practical application, resource allocation, and enforcement at the regional level.”* | **Intro** |
| **Method** | |  |  |
| • The HTA survey was administered to 9 organizations of HTA in the country but the total number of organizations working in HTA in the country is unclear – ie. How many are there in Switzerland so the reader can understand how representative this sample is so please include. Additionally later it talks about 13 institutions – please clarify the numbers. | | Thank you for the helpful comment. Extensive consultations with local and national experts in Swiss HTA highlighted that the HTA community in Switzerland is relatively small, with only a limited number of organizations actively involved. The authors also consulted the Swiss Network for HTA (SNHTA), the national network comprising most HTA organizations and entities (members) operating in Switzerland. The official list of member institutions can be found here: <https://www.snhta.ch/member-institutions.html>.  The number of members in this network is small, and our study included most of them through both the survey and interview tools. Additionally, we consulted the WHO HTA unit in Geneva for accurate information on Swiss HTA organizations, confirming this relatively small number of Swiss HTA organizations.  Based on these extensive international and local consultations supported by a literature review, the study methodology was designed to target up to ten existing local and national HTA organizations. Of the thirteen organizations invited, nine responded.  The relevant paragraphs in the Methods section, particularly under “Study Population” and “Sampling Strategy,” have been amended to reflect this information. Both paragraphs now read as follows:  *“The first group included nine of the thirteen invited HTA-associated organizations operating in Switzerland’s health sector, representing governmental, academic, private, and non-governmental (NGO) organizations.”*  *“The thirteen selected organizations were invited to participate, and nine agreed. Their representatives were then asked to nominate or assign team members involved in technical and operational HTA processes within their organizations to complete the survey.”* | **Methods** |
| • Also to confirm there were 8 in depth interviews with 9 experts – does that mean 2 were done together, were they from the same organization? Please clarify. | | The authors have revised the sections on IDIs in the Methods and added the requested clarifications. To clarify this, 10 experts from the same selected HTA organizations were invited, and eight experts (out of ten) accepted to participate in individual IDI. Relevant paragraphs, particularly under “Study Population,” “Sampling Strategy,” and “Data Collection,” have been amended to reflect these updates. The paragraphs below provide a sample of the revised Methods sections, which now read as follows:  *“For the IDI,* *ten high-level experts from the same HTA organization representing various sectors were invited, and eight participated. The interviews focused primarily on policy-related aspects of HTA.”*  *“The second group comprised ten experts/ leaders from these organizations responsible for overseeing HTA policies and strategic issues. All were invited, and eight participated in individual IDIs.”* | **Methods** |
| • Otherwise, the methods sound robust – when WHO also does a situational analysis of HTA in a country we do a literature review, use the survey as well as do in-depth interviews to get a complete picture. | | We thank the reviewer for the positive feedback. Our approach, combining literature review, surveys, and in-depth interviews, follows WHO practices to provide a comprehensive view of the Swiss HTA landscape. |  |
| **Results** | |  |  |
| These appear to be comprehensive | | We thank the reviewer for the positive comment and are pleased that the results are considered comprehensive. |  |
| **Discussion** | |  |  |
| • This would benefit with some restructuring and reframing – maybe highlight the discussion elements under some sub headings for clarity of what you are trying to highlight, or focus on | | We thank the reviewer for this valuable suggestion. We have restructured and reframed the discussion section to improve clarity, organizing the key discussion elements under subheadings that reflect the main themes of our study, HTA system pillars. This approach now clearly highlights stakeholder understanding, governance and regulation, resources and capacity, HTA implementation and utilization, evaluation of health technologies, and gaps with proposed solutions, allowing readers to follow the discussion more effectively. | **Discussion** |
| • Additionally, once again maybe putting into the context of HTAR – highlighting how the JCAs coming from the EU level could be considered by national or canton level organizations. | | Thanks for highlighting this important comment. The authors have added an additional discussion paragraph on HTAR and JCAs in the context of HTA in Switzerland. This discussion paragraph now reads as follows:  *“The coherence and integration of EU-level JCAs into Switzerland’s national and canton-level HTA processes presents both opportunities and challenges. On one hand, JCAs provide a robust and standardized evidence base that can support Swiss HTA bodies, such as the FOPH and cantonal health authorities, in streamlining local assessments, reducing duplication of work, and accelerating decision-making. This is particularly valuable for smaller cantonal HTA offices or specialized committees that may have limited resources to conduct full clinical evaluations independently. On the other hand, Swiss HTA organizations must carefully consider national healthcare priorities, population characteristics, and the regulatory and reimbursement frameworks unique to Switzerland when interpreting and applying JCA findings. Adapting EU assessments to the Swiss context ensures that recommendations remain relevant and actionable, but may require additional methodological adjustments or supplementary analyses, such as incorporating local cost-effectiveness data or national clinical practice patterns (2 reference added). Overall, leveraging JCAs can enhance efficiency and consistency in Swiss HTA while maintaining the flexibility necessary to address the specific needs of both federal and cantonal healthcare systems.”* | **Discussion** |
| **Section B: Reviewer 2 Comments** | | | |
| This is a small preliminary study regarding the HTA landscape in Switzerland. The authors note this limitation. I would be interested to know why the authors chose to conduct this study. It is important to spread the word about the importance of HTA in decision-making however I question what this article will provide for others who may be setting up HTA in their country, as there are always different contexts. I didn’t find the article particularly well written (although acceptable) and some statements were made without any reference to evidence e.g. the statement about registries and their limited quality and utility. There was a process described for identifying organisations to survey but it really wasnt very clear e.g. research members talking to collaborators they knew in Switzerland. | We thank the reviewer for these constructive comments. The rationale for conducting this study was to:   - Address a clear gap in the literature on HTA in Switzerland from policy and system perspective - HTA landscape and system pillars in Switzerland remains insufficiently described or assessed - Limited previous study has systematically examined the HTA system pillars, including stakeholder understanding, governance, resources, and HTA implementation across national and canton-level bodies. - The first national study using system analysis and mixed methods approach. - The study is added value because it generates this important foundational evidence not only for informing ongoing Swiss HTA reforms but also for offering insights to other countries with similarly decentralized systems.   All these points related to rational of conducting this study are clearly highlighted in the introduction section before the study aim and objectives.  We appreciate the reviewer’s observation regarding clarity and referencing. In response, we have revised and edited all manuscript’s sections to ensure it is strengthened by editing it grammatically, adding supporting clarification, explanation, and discussion, adding new references, especially for statement registries and their limited quality and utility, and other statements. Overall clarity of the writing is now further improved.  We have also revised the methods sections, and we added additional information to more clearly describe the methodology process, especially the extensive consultation with local collaborators, which is part of two methods were used along with the literature review to explore and identify names and information (lists) of main active organizations and individuals (experts/leaders). We are grateful for the reviewer’s feedback, which has helped improve the quality and clarity of this method section. | | **Intro and methods** |
|  |  | |  |
